# Recent Advances on PGPR, Beneficial Fungi, and Nanoparticles in Plant Abiotic Stress Tolerance: Current Evidence, Emerging Interactions, and Research Gaps

**DOI:** 10.3390/plants15213458

**Published:** 2026-11-05

**Authors:** Sajida, Hamdy Kashtoh, Tensangmu Lama Tamang, Kwang-Hyun Baek

**Affiliations:** 1 Department of Botany, Hazara University, Mansehra 21300, Pakistan; 2 Department of Biotechnology, Yeungnam University, Gyeongsan 38541, Gyeongbuk, Republic of Korea; 3 School of Chemical Engineering, Yeungnam University, Gyeongsan 38541, Gyeongbuk, Republic of Korea

**Keywords:** abiotic stress, PGPR, beneficial fungi, nanoparticles, sustainable agriculture

## Abstract

Various abiotic challenges, including severe drought, extreme temperatures, salinity, and heavy metal toxicity, substantially limit plant growth and agricultural sustainability on a global scale. Sustainable approaches that strengthen crop resilience while minimizing environmental impacts are therefore urgently needed. Plant growth-promoting rhizobacteria (PGPR), beneficial fungi, and nanoparticles have individually demonstrated considerable potential in improving plant growth, nutrient acquisition, antioxidant defense, and stress tolerance. PGPR enhance plant performance through phytohormone production, nutrient solubilization, 1-aminocyclopropane-1-carboxylate (ACC) deaminase activity, and activation of systemic defense responses. Beneficial fungi augment water uptake, nutrient absorption, osmotic adjustment, and stress-responsive signaling pathways, whereas nanoparticles facilitate efficient nutrient delivery, enhance photosynthetic performance, and support plant adaptation under adverse environmental conditions. Recent studies have further highlighted the synergistic benefits of PGPR–fungi, fungi–nanoparticle, and PGPR–nanoparticle interactions in mitigating abiotic stress. However, integrated tripartite interactions among PGPR, beneficial fungi, and nanoparticles remain insufficiently explored. This review summarizes current knowledge regarding their individual and combined roles, discusses the underlying physiological and molecular mechanisms, identifies existing research gaps, and proposes future perspectives for developing climate-resilient sustainable agricultural systems.

## 1. Introduction

Rapid global population growth has caused a surge in food consumption, placing mounting pressure on agricultural systems to produce higher crop yields without compromising crop quality and sustainability [1]. Meanwhile, global warming has intensified the frequency, severity, and persistence of abiotic stresses, posing adverse effects on crop production and further threatening global food security [2]. Among these stresses, drought, temperature extremes, salinity, and heavy metal toxicity are major environmental constraints that disrupt plant growth-related physiological and biochemical processes, causing substantial losses in crop yield [3]. Simultaneously, the excessive and inefficient use of chemical fertilizers has caused a decline in soil health, creating an additional challenge for agricultural sustainability [4]. For instance, stress-impaired root nutrient uptake and nutrient immobilization can further reduce fertilizer-use efficiency [5]. Under these conditions, the application of microbial inoculants is gaining prominence as an alternative to conventional chemical fertilizers, offering the potential to enhance agricultural productivity while conserving soil health and ensuring food safety [5]. These approaches are relevant to Sustainable Development Goals 2 (Zero Hunger), 12 (Responsible Consumption and Production), and 13 (Climate Action); however, their contribution depends on achieving tangible stability in productivity, savings in inputs, and environmental safety rather than merely assuming sustainability.

Plant-associated microorganisms, particularly plant growth-promoting rhizobacteria (PGPR) and beneficial fungi, play a vital role in promoting plant growth, nutrient uptake, and adaptation to environmental stresses [6]. These microorganisms build beneficial interactions with plants and regulate root development, nutrient absorption, and physiological processes by producing a diverse array of bioactive substances, including phytohormones such as indole-3-acetic acid (IAA), enzymes such as 1-aminocyclopropane-1-carboxylate (ACC) deaminase and phosphatases, siderophores, and various secondary metabolites such as microbial volatile compounds, thereby promoting plant growth and stress tolerance [7]. Furthermore, they improve soil fertility and quality by influencing its structure, nutrient cycling, and organic matter decomposition, facilitating plants’ access to water and essential nutrients [8]. In addition, microbial colonization can activate plant defense mechanisms and antioxidant systems, thereby increasing plants’ tolerance to abiotic stress [7]. Consequently, microbial inoculation is gaining increasing attention as an approach within eco-friendly sustainable agricultural technologies to enhance plant growth and yield under adverse conditions [9,10].

Despite the promising benefits of PGPR and beneficial fungi, their effectiveness is often limited by poor survival rates, inefficient root colonization, and may differ according to soil properties, microbial compatibility, and the severity of abiotic stress [11]. To address these challenges, complementary approaches such as nanotechnology are increasingly recognized as promising strategies for enhancing plant stress resilience and strengthening beneficial plant–microbe interactions [12]. Engineered nanomaterials can enhance nutrient availability, improve moisture retention, and increase the efficacy of agrochemical delivery through controlled-release systems, thereby reducing environmental impacts [13]. Moreover, nanomaterials have been reported to support plant hormonal regulation and alleviate the negative effects of environmental stresses, making them valuable components of modern sustainable agriculture [14]. The combined use of beneficial microorganisms and nanomaterials offers a complementary approach for strengthening plant resilience, crop production, and agricultural sustainability [15,16].

**Figure 1:**
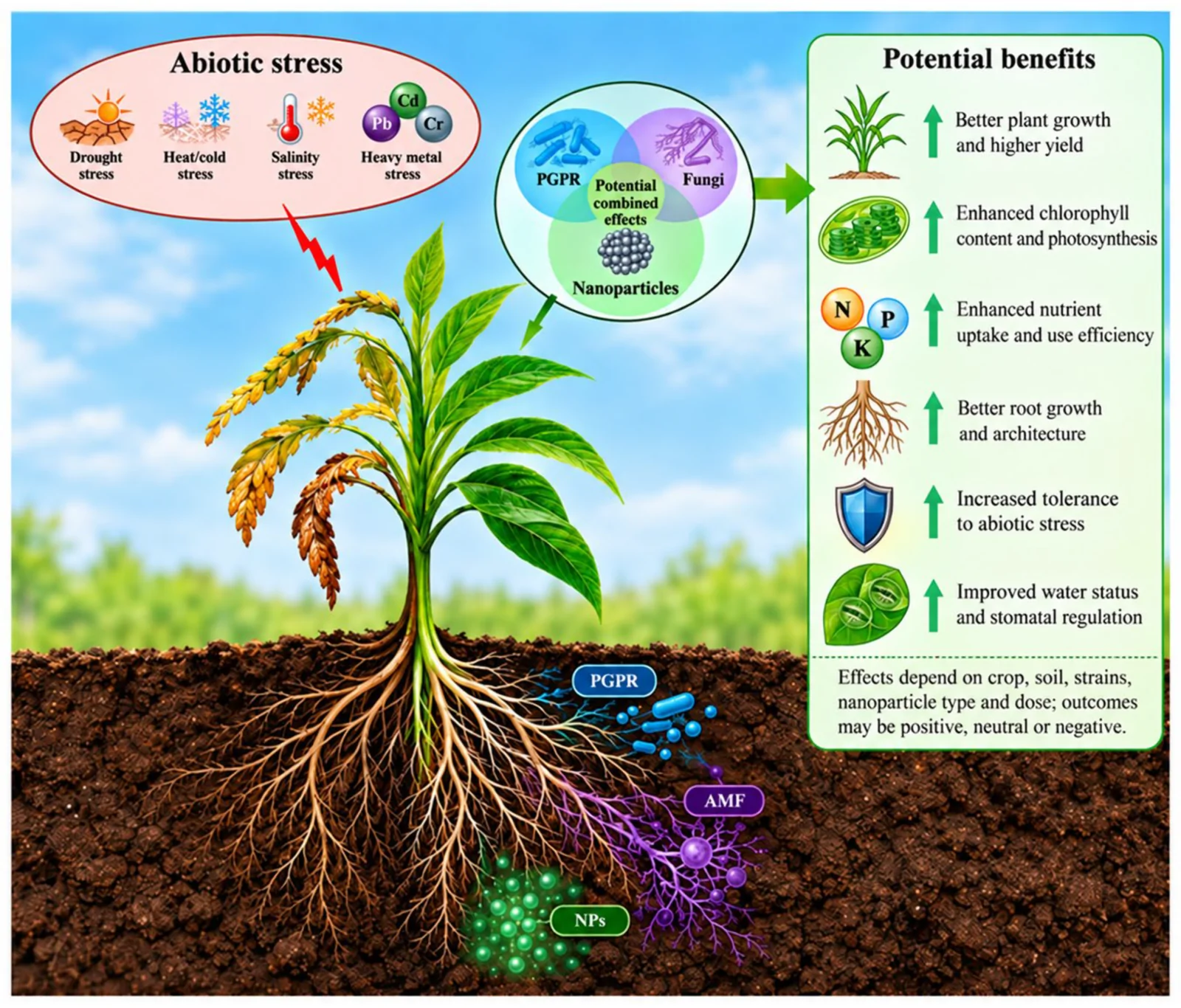
Schematic diagram showing PGPR, fungi including arbuscular mycorrhizal fungi (AMF), and nanoparticles to support plant performance under abiotic stresses such as drought, extreme temperature, salinity, and heavy-metal exposure (Pb = lead, Cd = cadmium, and Cr = chromium). Depending on the crop, soil conditions, microbial strains, nanoparticle type, and dose, these treatments enhance plant tolerance to abiotic stresses. The symbols ↑ indicate increases/enhancement in the corresponding parameters.

Recent studies have increasingly focused on the interactions between plants, beneficial microorganisms, and nanomaterials as a potential approach to boost plant growth and acclimation ability to abiotic stresses [12,17,18]. This integrated system enhances nutrient acquisition, strengthens plant defense mechanisms, improves water-use efficiency, and reduces oxidative damage under stressful environmental conditions. Beneficial microorganisms regulate phytohormone balance and support stress-responsive pathways, while nanomaterials facilitate the efficient delivery of microbial inoculants and improve rhizosphere colonization. Collectively, these interactions contribute to enhanced plant development, adaptability, and productivity under challenging stress conditions (Figure 1) [19,20,21].

Although several studies have assessed the individual roles of PGPR, mycorrhizal fungi, and nanoparticles in promoting plant growth and abiotic stress tolerance, limited attention has been given to their integrated or tripartite interactions. Current research mainly focuses on plant–microbe or plant–nanoparticle relationships, while the synergistic mechanisms involving PGPR, fungi, and nanoparticles remain insufficiently understood. Therefore, a comprehensive review is needed to summarize prevailing knowledge and identify future research directions regarding their combined application under abiotic stress conditions.

This review aims to evaluate current knowledge of the individual and combined roles of PGPR, mycorrhizal fungi, and nanoparticles in promoting plant growth and abiotic stress adaptation. Unlike previous reviews, the present review article provides an integrated overview of the binary interactions between PGPR and mycorrhizal fungi, PGPR and nanoparticles, and mycorrhizal fungi and nanoparticles, while also highlighting the potential combined effects of all three components, with particular emphasis on their impacts on plant growth and responses to abiotic stresses. Accordingly, this review aims to synthesize the mechanistic and physiological evidence for these interactions, assess the strength and limits of binary and tripartite treatment evidence, and identify requirements for field validation. Furthermore, the review addresses the limited existing knowledge of tripartite interactions among these microorganisms and nanoparticles, identifies current research gaps, and proposes future research perspectives for their potential integration in sustainable agriculture.

## 2. Effects of Abiotic Stress on Plant Growth and Development

### 2.1. General Effects of Abiotic Stress

Abiotic stresses are critical environmental constraints limiting agronomic growth and productivity. For optimal development, plants require adequate water, light, and favorable temperatures; however, harsh environmental determinants such as drought, temperature extremes, salinity, and heavy metal toxicity disrupt normal physiological and biochemical processes [22,23]. Abiotic stresses negatively modulate seed germination and early seedling establishment by delaying germination, reducing germination percentage, decreasing seedling vigor, and suppressing root and shoot growth [24]. These stresses can induce morphological alterations, including stunted plant height, reduced leaf area, and impaired root development, disrupting water and nutrient balance [25]. Moreover, they disrupt cellular metabolism and homeostasis, membrane stability, gene expression, and photosynthetic efficiency, culminating in substantial declines in plant growth, biomass accumulation, and agricultural yield [26]. Therefore, these abiotic stresses represent major constraints to successful crop establishment and productivity.

### 2.2. Effects of Drought Stress

Drought stress is a major abiotic constraint that poses a substantial risk to agricultural productivity and global food security. It causes water deficits that reduce plant water potential, promoting stomatal closure and restricting the acquisition and translocation of water and essential nutrients [27]. As a result, photosynthesis, nutrient uptake, and oxidative homeostasis are severely impaired, inducing hydraulic failure, causing leaves to wilt, curl, or turn yellow, and may eventually result in plant death because of permanent cellular damage caused by prolonged drought [28].

Drought stress affects plants throughout their developmental stages, from seed germination and root growth to floral emergence and grain filling [29]. Especially during flowering, fruit set, and grain filling, water deficits disrupt carbohydrate partitioning and assimilate transport, resulting in reduced flower production, poor fruit development, impaired grain filling, and yield losses [30]. Furthermore, it also limits leaf expansion, reduces fresh biomass, and limits the accumulation of sugars, nutritional components, and flavor-associated metabolites [31,32]. These effects compromise the economic and nutritional quality of crops, causing financial losses in agricultural production and contributing to food scarcity.

During drought stress, plants respond primarily by closing their stomata to minimize water loss. However, this defense response also decreases carbon dioxide (CO_2_) intake, which directly affects the rate of photosynthesis and transpiration [33,34]. In addition, drought also suppresses chlorophyll biosynthesis and accelerates its degradation, further limiting the rate [35,36]. Drought stress-induced oxidative damage results in membrane lipid peroxidation, causing higher membrane permeability and rapid electrolyte leakage, leading to structural and functional damage to plant cells [37,38].

### 2.3. Effects of Salt Stress

Soil salinity is another major constraint limiting sustainable agricultural production across the world [39]. Although salt tolerance varies considerably among various plant species, with some species being more susceptible to saline conditions than others, salt stress can still hamper plant growth and development [40]. Initially, salt stress induces osmotic stress, lowering soil water availability. This limits water and nutrient uptake and leads to physiological drought, which inhibits seed germination, cell expansion, lateral root formation, and overall plant growth [41,42]. Prolonged salinity stress increases the accumulation of sodium (Na^+^) and chloride (Cl^−^) ions in plant tissues, causing ionic toxicity and nutrient imbalance [43]. Upon translocation of these ions from roots to shoots, they interfere with enzyme activity, membrane function, photosynthesis, and respiration [44,45]. As a result, these physiological disruptions hinder plant development, including slower seedling growth, leaf chlorosis, root stunting, and may also cause abnormal shoot growth [46,47,48].

Similarly, salinity stress disrupts osmotic balance and ion homeostasis, promoting excessive generation of reactive oxygen species (ROS) [49]. Overproduction of ROS causes oxidative damage, including lipid peroxidation, disruptions in membrane and ion transport, protein oxidation, DNA damage, alterations in gene transcription associated with stress responses, and ultimately cell death [50,51]. Plants counteract ROS through enzymatic (catalase, ascorbate peroxidase, and superoxide dismutase) and non-enzymatic (ascorbate and glutathione) antioxidants. However, severe and prolonged salinity stress can overwhelm these antioxidant defense mechanisms, necessitating stress-mitigating strategies to boost plant resilience to salt stress [52].

### 2.4. Effects of Other Abiotic Stresses

Besides drought and salinity, temperature extremes are other pronounced abiotic stresses that adversely affect the production and quality of crops [53,54]. Global warming has intensified the occurrence and severity of both heat and cold stress, creating significant challenges for sustainable agriculture. Extreme temperatures, such as low temperatures, inhibit germination and water uptake, whereas high temperatures may induce dormancy and restrict seedling growth [55,56]. High temperatures severely affect crop production, especially during pollination and early grain development [57]. When temperature exceeds 33–35 °C, the viability of pollen, fertilization efficacy, and seed production declines in crops like maize, rice, sorghum, and soybean, with effects intensified by high vapor pressure deficit [58,59,60,61]. Furthermore, heat stress can disrupt cell division, endosperm growth, and grain filling, resulting in grain size reduction and low yield.

Temperature stress disrupts protein structures and membrane lipids, disrupting normal physiological metabolism. Especially in vegetable crops, which are more sensitive to prolonged high temperatures, this reduces photosynthetic efficiency and chlorophyll accumulation, ultimately restricting plant growth and productivity [62,63]. Besides high temperature stress, plants can also experience physiological disorders, such as surface depression and internal browning under cold stress, particularly in root crops exposed to temperatures below 10 °C [64]. It also alters plant metabolism by promoting starch degradation and sugar accumulation, while interfering with volatile organic compound production and fatty acid metabolism, consequently altering the flavor, aroma, and overall crop quality [65].

Heavy metal pollution is another important environmental stress that negatively affects plant growth. Unlike transient stresses, heavy metals tend to persist and accumulate in soil and plant tissues, interfering with essential elements such as calcium, iron, zinc, and magnesium [66,67]. Heavy metal toxicity can hinder germination and early plant development by interfering with nutrient uptake, photosynthesis, cellular metabolism, and inducing ROS-mediated oxidative stress, resulting in damage to cellular components [68,69,70]. Furthermore, prolonged heavy metal exposure can affect plant–microbiome interactions, impeding nutrient cycling. Heavy metals can also accumulate in tissues of edible plants and enter the human food chain, posing a serious risk to human health, including chronic toxicity and irreversible damage to several organs [71,72]. Thus, excessive heavy metal accumulation in plants not only reduces agricultural product quality but also threatens food safety and human health. Collectively, abiotic stresses induce various physiological and biochemical effects that adversely affect plant growth and yield.

## 3. PGPR in Enhancing Plant Growth and Abiotic Stress Tolerance

PGPR are root-colonizing bacteria that promote plant growth and enhance resilience through nutrient assimilation, hormone regulation, stress management, and pathogen suppression, making them significant bio-agents for sustainable agriculture [8]. Many PGPR genera comprising *Azotobacter*, *Azospirillum*, *Bacillus*, *Burkholderia*, *Flavobacterium*, *Pseudomonas*, *Rhizobium*, and *Xanthomonas* have been shown to play a vital role in plant growth promotion and stress tolerance [73,74,75,76]. These bacteria facilitate the translocation of water and essential nutrients, thereby boosting nitrogen, phosphorus, and potassium acquisition and promoting optimal plant growth and development [77]. They also influence rhizosphere dynamics, modifying root exudates and recruiting beneficial microbial communities, ultimately supporting the formation of a stress-resilient plant microbiome [78].

Several research studies have shown the effectiveness of PGPR in improving plant tolerance to adverse abiotic stresses and enhancing growth. For instance, PGPR isolates such as *Streptomyces* sp. KhEc 44, *Bacillus paralicheniformis* KhEc 68, and *Priestia filamentosa* KhEc 69 were able to grow under high salinity, alkaline pH, and high temperature, indicating their multi-stress tolerant characteristics. These inoculants significantly enhanced growth and salt stress tolerance of mung bean [79]. *Pseudomonas* sp. M25 enhanced drought tolerance in *Arabidopsis* primarily by increasing root hair density and elongation [80]. Likewise, *Acinetobacter pittii* enhanced cadmium stress tolerance by regulating genes associated with bacterial migration and carbon and amino acid metabolism, while promoting cadmium sequestration in the rhizosphere [81]. Similarly, *Bacillus velezensis* KB21 promoted cucumber growth and enhanced salt tolerance by boosting antioxidant activity, preserving Na^+^/K^+^ homeostasis, and modulating genes related to stress response and abscisic acid [82]. Moreover, genomic analysis of *Bacillus glycinifermentans* isolated from date palm revealed multiple functional traits associated with plant growth promotion. Upon inoculation on seeds of *Hordeum vulgare* L., it significantly improved seed germination and seedling growth, and reduced superoxide dismutase activity compared to non-inoculated seeds under copper stress [83]. These findings collectively emphasize the pronounced role of PGPR in enhancing plant growth, nutrient acquisition, and resilience against multiple abiotic stresses.

PGPR positively influence plant growth under various abiotic stresses through multiple direct and indirect mechanisms, collectively contributing to induced systemic tolerance [84,85]. One of the key mechanisms involves improved nutrient acquisition through biological nitrogen fixation, phosphorus and potassium solubilization, and iron acquisition mediated by siderophores [86]. Moreover, PGPR influence plant growth by modulating phytohormone levels such as auxins, cytokinins, and gibberellins, while also reducing stress-induced ethylene levels through the activity of ACC deaminase [87,88]. These hormonal effects promote cell growth and division under adverse abiotic stress.

PGPR further enhance plant stress tolerance through osmoregulation and activation of the antioxidant defense system. PGPR-produced exopolysaccharides improve soil aggregation and moisture retention [89]. They also increase the accumulation of chlorophyll, carotenoids, proline, and other osmoprotectants, thereby maintaining photosynthesis efficiency and cellular stability under water deficit conditions [90]. Furthermore, PGPR regulates the antioxidant defense system by reducing ROS generation and maintaining cellular redox balance [91]. For instance, PGPR strains, such as *Bacillus amyloliquefaciens* NBRISN13, demonstrated remarkable potential in enhancing antioxidant defense and improving carbon and nitrogen metabolism [92,93]. Similarly, *Bacillus amyloliquefaciens*, *Pseudomonas putida*, and *Pseudomonas fluorescens* mitigated heavy metal toxicity in rice by scavenging ROS and minimizing oxidative damage [94].

PGPR induce plant defense responses through molecular signaling pathways, including the release of microbial volatile organic compounds, phytohormone signaling, and gene expression [95]. Through these signaling interactions, PGPR coordinate with specific plant receptors to activate transcription factors that are involved in key developmental processes and adaptive responses to abiotic stresses [96]. For example, auxins synthesized by PGPR bind to plant receptors and trigger auxin-responsive factors, which regulate genes involved in root growth and elongation [97]. Similarly, cytokinins produced by microbial partners interact with membrane-bound receptors, leading to the activation of signaling cascades that enhance cell division while delaying the onset of senescence, especially under adverse environmental conditions [98]. In addition, PGPR can modulate ethylene levels in plants, thereby affecting ethylene-responsive transcription factors and reducing stress-induced damage [99]. PGPR such as *Bacillus velezensis* KB21 upregulated genes encoding Na^+^/H^+^ antiporters (NHX), high-affinity K^+^ transporters (HKT), and aquaporins (AQPs), highlighting its role in maintaining ion homeostasis during salinity stress [82]. PGPR also regulate key drought response genes, such as *DREB2A*, *ERF1*, *WRKY70*, *NCED1*, and *P5CS*, which regulate abscisic acid-mediated signaling, osmotic adjustment, and stress adaptation [100].

Collectively, these PGPR-induced physiological and molecular responses contribute to improved nutrient utilization, enhanced plant growth, and greater resistance to adverse environmental challenges, thereby enhancing plant survival and increasing crop productivity and quality under abiotic stresses.

## 4. Beneficial Fungi in Plant Abiotic Stress Management

Beneficial fungi represent a diverse consortium of microorganisms that associate with plant roots through symbiotic associations, facilitating plant growth and strengthening tolerance to unfavorable environmental conditions. Among beneficial fungi, Arbuscular mycorrhizal fungi (AMF) are highly prevalent fungi forming symbiotic associations with about 72% of vascular plant species [101,102]. Various studies have demonstrated that these fungi improve nutrient acquisition, particularly phosphorus and nitrogen, enhance water absorption, and boost plant resilience to adverse environmental conditions such as drought, salinity, and waterlogging [103,104].

AMF form extraradical hyphae that expand the surface area of the root system and extend beyond the root depletion zone, thereby enhancing uptake of nitrogen and potassium, as well as improving access to poorly mobile nutrients, such as phosphorus [105,106]. Moreover, they form mycorrhizal networks connecting multiple plant species and facilitating nutrient exchange between them. AMF also regulate soil nutrient cycling and enhance nutrient availability through interactions with soil microorganisms and organic matter degradation, thereby sustaining soil fertility and overall plant growth [107,108].

Besides promoting root functions and soil fertility, AMF also alleviate abiotic stress by enhancing photosynthetic efficiency, promoting plant biomass accumulation, and modulating phytohormone function and signaling [109]. For instance, inoculation of *Rhizophagus irregularis* in tomato plants exposed to salinity influenced the interaction between ethylene and gibberellin levels, contributing to improved plant growth [110]. This suggests that AMF-mediated hormonal crosstalk plays a pivotal role in maintaining plant resilience to salinity stress. Similarly, AMF regulate complex signaling pathways and gene transcription within host plants, resulting in enhanced physiological performance, gas exchange, and accelerated plant growth under adverse abiotic stress [111]. For example, AMF compound inoculants resulted in a significant increase in the net CO_2_ assimilation rate (*A*) in rice seedlings exposed to salinity stress, indicating an improvement in photosynthetic performance under conditions of moderate salt stress [112]. Moreover, AMF inoculation showed higher *OsQHB* and its related *PRX* genes expression, which can contribute to enhanced ROS-scavenging enzyme activity. Similarly, in drought-stressed grapevine, AMF increased proline accumulation and upregulated *VvP5CS*, while also modulating abscisic acid-related genes such as *VvNCED* [113]. In addition, changes in aquaporin gene expression may contribute to improved plant water balance under drought stress, while certain plasma membrane intrinsic proteins (PIPs) may also regulate membrane CO_2_ permeability and mesophyll conductance, thereby influencing photosynthetic CO_2_ diffusion. The expression of aquaporin genes was also affected, which may contribute to improved water balance and CO_2_ homeostasis during drought stress.

Overall, mycorrhizal fungi constitute a vital component of strategies for mitigating plant stress and play a significant role in fostering long-term agricultural sustainability. However, intensive agricultural practices can negatively affect these beneficial interactions, potentially reducing their effectiveness. Therefore, adopting sustainable agricultural methods is integral to preserving these symbiotic relationships [114,115].

## 5. Role of Nanoparticles in Sustainable Agriculture

Conventional agricultural practices alone are increasingly insufficient to meet the growing requirements of food production under changing environmental conditions [116]. Therefore, the design and development of innovative, effective, and sustainable technologies has become essential, with nanotechnology emerging as a promising avenue for addressing these challenges. As one of the key scientific advancements of the 21st century, nanotechnology has gained significant importance across multiple sectors, including agriculture, medicine, food technology, environmental monitoring, water purification, and electronics [117,118,119]. In recent years, there has been significant expansion in the application of nanotechnology in plant science, particularly in promoting the ability of plants to tolerate various abiotic stresses [120].

Research studies have investigated the potential of nanomaterials to enhance plant growth, physiological performance, and stress resilience, emphasizing their significance in sustainable agricultural systems [121,122,123]. For instance, the application of selenium nanoparticles alleviated cadmium-induced growth inhibition and oxidative damage in mung bean. The optimal selenium nanoparticles treatment enhanced plant growth, biomass, and photosynthetic parameters, increased soluble proteins, and reduced proline and malondialdehyde (MDA) levels [124]. In addition, selenium nanoparticles reduced cadmium accumulation in leaves and grains, thereby mitigating cadmium stress. Similarly, foliar spray of calcium nanoparticles to canola plants improved mineral absorption, chlorophyll content, mesophyll ultrastructure, gas exchange, and photosystem I (PSI) and II (PSII) efficiency [125]. Furthermore, it enhanced photoprotection of PSII during drought through heat dissipation. Treatment of wheat plants with zinc oxide (ZnO) and titanium dioxide (TiO_2_) nanoparticles significantly enhanced heat stress tolerance through an increase in superoxide dismutase and glutathione peroxidase activity, lowering hydrogen peroxide (H_2_O_2_) levels, thereby stabilizing membranes [126].

Nanoparticles can enter plant tissues by penetrating the cell wall, with roots serving as one of the major routes for nanoparticle uptake. Their uptake by plants is significantly affected by particle size, surface properties, and coating materials, as these factors affect absorption efficiency and the rate of accumulation within plant cells [127]. Entry of nanoparticles into plant cells may occur via formation of temporary pores, carrier-mediated transport, or interaction with membrane proteins such as aquaporins and ion channels. Increasing evidence suggests that endocytosis-like mechanisms play a major role in the uptake of nanomaterials into plant cells. Consequently, it influences various physiological processes such as nutrient uptake, stomatal conductance, photosynthesis, redox homeostasis, and root and shoot growth [128,129]. These interactions can also regulate genes related to essential pathways orchestrating plant growth, development, and adaptation to environmental stresses by interacting with DNA or RNA [130,131].

Nanoparticles can serve as priming agents or nanocarriers for nutrients, enabling plants to cope with multiple abiotic stressors while promoting their growth. However, concerns related to nanoparticle toxicity as well as environmental and food safety cannot be overlooked. Therefore, advancement and regulated application of biodegradable nanoparticles should be promoted to foster sustainable agriculture.

## 6. Complementary-Synergistic Interaction Between Beneficial Fungi, PGPR, and Nanoparticles

**Figure 2:**
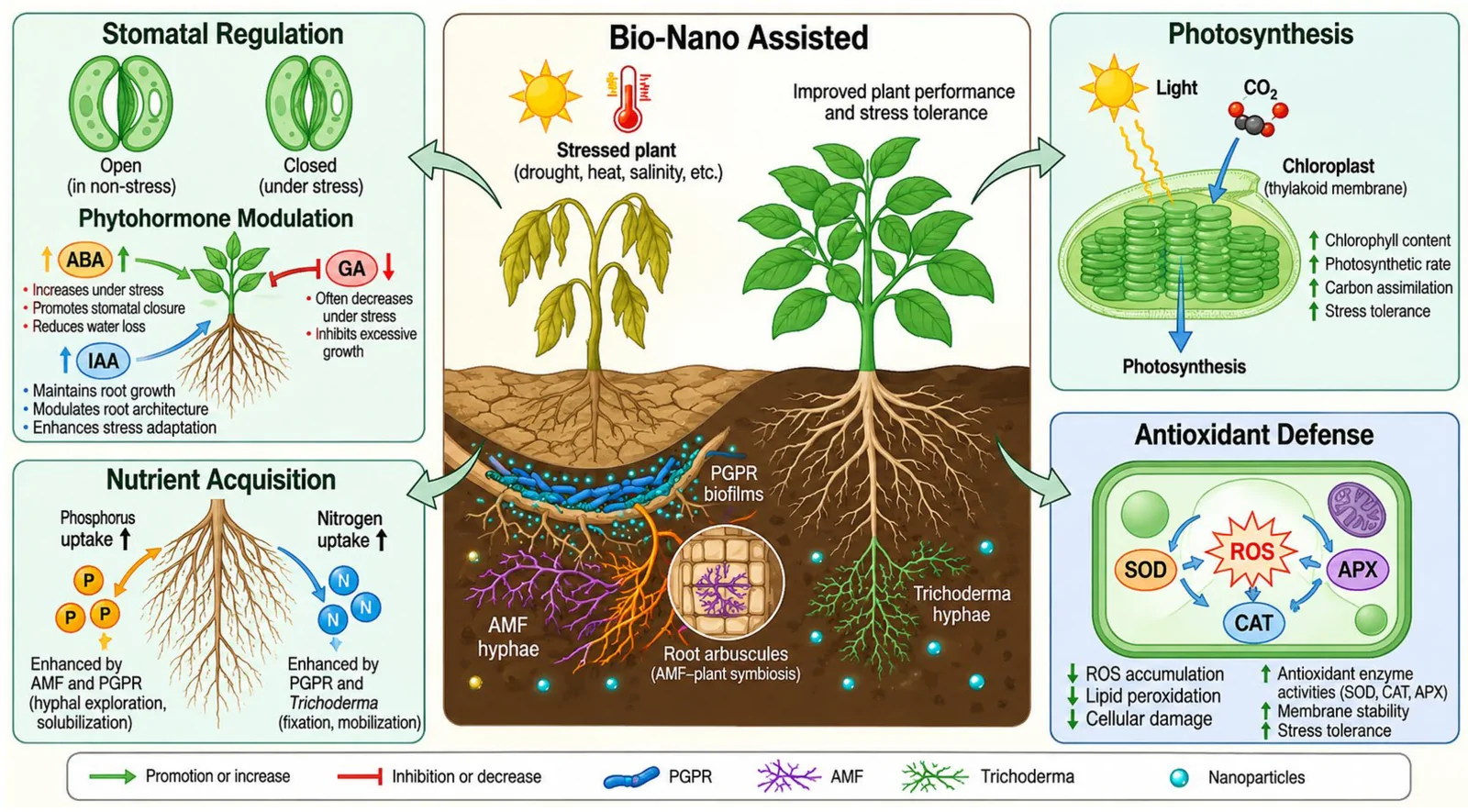
Schematic diagram of bio-nano assisted proposed mechanisms for abiotic stress tolerance in plants. Plants inoculated with PGPR/beneficial fungi/nanoparticles exhibit growth-promoting traits by enhancing physiological traits, reducing oxidative stress, improving beneficial fungi colonization, nutrient acquisition, photosynthesis, and stomatal regulation. These proposed mechanisms promote plant growth and abiotic stress tolerance. The symbols ↑ and ↓ indicate increases and decreases, respectively.

Integrating beneficial microorganisms with nanomaterials represents a promising nano-bio-based approach for enhancing plant growth and resilience to abiotic stresses. Beneficial bacteria and fungi facilitate nutrient uptake, participate in phytohormone signaling, and colonize the rhizosphere to activate plant defense responses [132,133]. In the meantime, depending on the properties and application method, nanoparticles can increase nutrient availability, water uptake, and regulate physiological functions, while also enabling the targeted delivery and controlled release of agrochemicals [134,135]. Their combined use can yield complementary or synergistic effects by promoting microbial colonization and activity, improving nutrient and water use efficiency, and strengthening antioxidant and defense mechanisms under adverse abiotic stresses. An overview of bio-nano-assisted mechanisms for abiotic stress resistance in plants is presented in Figure 2.

### 6.1. Fungi and PGPR

The co-application of beneficial fungi and PGPR has gained considerable attention as a sustainable strategy for boosting plant growth and tolerance to abiotic stresses. Beneficial fungi, including AMF, endophytic fungi, and other plant-associated fungal species, act synergistically with PGPR to improve nutrient cycling, increase nutrient availability [136], suppress soil-borne pathogens [137], and promote efficient nutrient uptake [138]. This microbial association also enhances soil aggregation and structure and establishes a favorable rhizosphere environment for plant development.

Several studies have reported that the co-inoculation of beneficial fungi and PGPR significantly increases plant biomass, improves plant stature, enhances the root-to-shoot ratio, and increases root dry biomass, while enhancing the activities of important soil enzymes, including urease, dehydrogenase, and sucrase [138,139]. These interactions improve soil fertility, microbial activity, and overall plant performance. Furthermore, co-inoculation enhances the degradation of environmental contaminants and strengthens plant defense mechanisms against abiotic stresses. For example, Begum et al. [140] demonstrated that the combined inoculation of AMF and PGPR in tobacco plants exposed to drought stress markedly improved plant growth, biomass accumulation, photosynthetic performance, chlorophyll content, and PSII efficiency. At the same time, the treatment reduced electrolyte leakage and lipid peroxidation while promoting the accumulation of phenolic compounds and flavonoids, thereby enhancing the antioxidant capacity and drought tolerance of the plants.

The co-application of AMF and PGPR) can alter nutrient dynamics within the “plant–fungus–bacterium” system, including carbon allocation and the cycling of phosphorus and nitrogen [141]. For instance, the phosphate-solubilizing bacterium *Rahnella aquatilis* has been shown to enhance phosphorus uptake and its subsequent transport through the hyphae of the fungus *Rhizophagus irregularis*; it was also associated with an increase in carbon transfer from the host plant *Medicago truncatula* to the fungus [142]. In maize, bacterial communities within the rhizosphere in collaboration with AMF facilitate the acquisition of insoluble phosphorus and potassium by the plant, suggesting that outcomes may depend on the specific composition of the indigenous bacterial community [143]. These interactions may not always be synergistic; however, a study has indicated that co-inoculation with bacteria and AMF most frequently results in cumulative plant growth responses [144]. Furthermore, these effects can vary depending on environmental conditions and the host plant. Carbon and phosphorus exchange vary among co-occurring plant species under different soil phosphorus levels [145], while drought intensity has been shown to alter fungal colonization responses and phosphorus uptake in co-inoculated maize plants [146]. Consequently, while competition for resources is a potential outcome, the observed variation in the biomass of root-associated fungi and bacteria linked to specific plant traits does not necessarily imply direct competition for carbon, nutrients, or specific soil micro-niches [147].

The beneficial effects of fungal–PGPR interactions have also been observed in several crops, including oat, soybean, and maize, where they improve nutrient acquisition, plant establishment, and resistance to drought, salinity, and other environmental challenges [140,148]. Therefore, the integrated use of beneficial fungi and PGPR represents an eco-friendly and sustainable approach for improving crop productivity, maintaining soil health, and supporting climate-resilient agriculture.

Although substantial evidence supports the synergistic use of beneficial fungi and PGPR for improving tolerance to drought and salinity, studies integrating these microbial partners with nanoparticles remain comparatively scarce. Current evidence is concentrated in a limited number of crops and stress-specific systems, highlighting the need for mechanistic and field-scale validation. Table 1 summarizes recent studies investigating the beneficial effects of combined application of beneficial fungi and PGPR in alleviating various abiotic stresses in different plant species and their mechanism of action.

### 6.2. Beneficial Fungi and Nanoparticles

Mycorrhizal fungi show a significant role in improving plant development and adaptation to abiotic stresses when used in combination with nanoparticles. Nanoparticles possess unique physicochemical properties, including a high surface-to-volume ratio, which enables the controlled and efficient transport of nutrients and agrochemicals, resulting in improved nutrient utilization and overall crop performance [164]. However, their long-term effects on soil microbial communities and ecosystem sustainability require further investigation [165].

Several studies have demonstrated that the combined application of beneficial fungi and nanoparticles influences physiological performance under stress conditions. For example, the inoculation of chili plants with beneficial fungi together with zinc and selenium nanoparticles increased chloroplast number and grana thickness, leading to improved photosynthetic capacity [166]. Similarly, the co-application of chitosan nanoparticles and beneficial fungi in wheat enhanced photosynthetic rate, stomatal conductance, PSII efficiency, and carbohydrate accumulation, resulting in better plant growth and stress adaptation [167]. The co-application of TiO_2_ nanoparticles and AMF improved nutrient status, water-use efficiency, and essential oil productivity in sage (*Salvia officinalis* L.) [168]. Beneficial fungi further improve plant resilience by modifying the rhizosphere environment, increasing nutrient solubility, enhancing mineral uptake, and strengthening antioxidant defense systems. These synergistic interactions improve photosynthetic efficiency, nutrient use efficiency, and overall plant adaptation to unfavorable environmental conditions while reducing dependence on conventional agrochemicals. Excessive ROS generation brought on by drought and salinity causes oxidative stress, which severely restricts plant growth and output. In drought- and salinity-stressed plants, iron oxide nanoparticles enhance redox regulation, iron homeostasis, and antioxidant defense, whereas AMF improves hormone balance, nutrient uptake, water absorption, and stress-responsive gene expression. The simultaneous application of iron nanoparticles and AMF generates synergistic effects on stress tolerance pathways. Combining physiological, molecular, and soil-based methods presents viable alternatives for stress-resilient, sustainable agriculture [169].

Although nanoparticles can promote plant growth at appropriate concentrations, excessive levels of metal-based nanoparticles such as Ag, TiO_2_, ZnO, and CuO may cause phytotoxicity by inhibiting seed germination, restricting root and shoot growth, damaging cell membranes, generating excessive ROS, disrupting photosynthetic pigments, and altering nutrient homeostasis. High nanoparticle concentrations may also reduce fungal colonization efficiency of beneficial fungi, including AMF, thereby weakening plant–fungus symbiosis and weakening plant performance and stress tolerance [170,171]. For instance, Ag nanoparticles and Fe_3_O_4_ nanoparticles have been shown to significantly reduce fungal colonization and decrease glomalin-related soil protein (GRSP) content, further indicating inhibitory effects on plant–fungus symbiosis [171,172]. Another study on maize found that exposure to ZnO nanoparticles reduced root colonization by AMF [173]. Another study on rice plants found that treatment with TiO_2_ nanoparticles resulted in a significant reduction in the formation of vesicles and arbuscules by AMF. Specifically, when using particles doped with 2 mol% TiO_2_, the root colonization rate decreased [174].

Despite these potential phytotoxic effects of nanoparticles, beneficial fungi have been widely reported to buffer plants against nanoparticle-induced toxicity by enhancing antioxidant defense mechanisms, improving mineral nutrition, regulating stress-responsive pathways, and maintaining cellular redox homeostasis [175,176,177]. These fungi help plants sustain growth and physiological functions even under nanoparticle exposure. [172,175,176,177]. Overall, beneficial fungi continue to provide significant protection in preserving plant development, physiological stability, and enhancing resilience against environmental challenges (summarized in Table 2), regardless of whether nanoparticles exert predominantly positive or negative influences on plant systems.

### 6.3. PGPR and Nanoparticles

PGPR and nanoparticles act complementarily or synergistically to enhance plant growth, productivity, and stress tolerance under adverse environmental conditions. PGPR enhance nutrient availability, phytohormone synthesis, and systemic resistance, while nanoparticles facilitate the efficient delivery of nutrients and agrochemicals, directly influencing plant physiological processes. Reports indicate that their combined use yields greater benefits than individual treatments, particularly under conditions of drought, salinity, and heavy metal stress [187]. Nevertheless, concerns regarding nanoparticle toxicity, long-term environmental impacts, and effects on soil microorganisms highlight the need for further research and the development of sustainable application strategies. Understanding the interactions among PGPR, nanoparticles, plants, and the soil microbiome will be essential for maximizing their agricultural potential while minimizing ecological risks [188].

The combined application of PGPR and nanoparticles has emerged as an effective strategy for improving plant growth and stress tolerance under diverse abiotic conditions [189]. Under drought stress, co-application enhances relative water content, stomatal conductance, photosynthetic pigment accumulation, nutrient uptake, and biomass production while reducing ethylene levels and electrolyte leakage. PGPR that possess ACC deaminase enzyme activity play a significant role in mitigating the accumulation of ethylene induced by drought. They achieve this by breaking down ACC, thereby reducing the growth inhibition caused by ethylene, which in turn promotes plant growth and enhances drought tolerance [179,190,191]. Under salinity stress, the synergistic interaction between PGPR and nanoparticles alleviates ionic toxicity, maintains osmotic balance, improves photosynthesis, and enhances lipid and protein metabolism, leading to better plant productivity than individual treatments [192]. Furthermore, PGPR–nanoparticle interactions contribute to heavy metal remediation by reducing the absorption and accumulation of toxic metals while promoting root development and aerial plant growth, and increasing biomass production in contaminated soils. For instance, the co-application of ZnO and TiO_2_ nanoparticles with the bacterium *Bacillus pumilus* improved the growth and physiological performance of maize, while simultaneously reducing the bioaccumulation of cadmium in its roots and stems under cadmium-stress conditions [193]. Similarly, ZnO nanoparticles combined with PGPR reduced cadmium uptake and bolstered antioxidant defenses in wheat [194]. In rice, the co-application of iron oxide nanoparticles with the bacterium *Bacillus megaterium* enhanced biomass production, photosynthetic performance, and nutrient uptake, while decreasing the accumulation of cadmium in plant tissues [195]. Similarly, integrated applications in ryegrass enhanced biomass while minimizing cadmium content compared with separate treatments [196,197]. Recent studies have also highlighted the benefits of combining TiO_2_ nanoparticles with PGPR, resulting in improved plant biomass, resilience, and productivity under drought, salinity, and pathogen stress [198]. Overall, the combined use of plant PGPR and nanoparticles represents a promising and sustainable approach to enhancing crop performance, tolerance to environmental stress, and agricultural productivity. Few examples of the complementary-synergistic use of PGPR and nanoparticles and their effect in enhancing plants’ stress tolerance are presented in Table 3.

Ahmadi-Nouraldinvand et al. utilized silicon dioxide nanoparticles in conjunction with AMF and PGPR inoculation on wheat to assess the co-effects of three components in mitigating the adverse impacts of salt stress [208]. Inoculating wheat with bio-fertilizers (PGPR and AMF) and silicon dioxide nanoparticles enhanced the physiological metrics of the wheat, including chlorophyll index, anthocyanin levels, leaf area index, and chlorophyll fluorescence parameters. It also elevated the concentrations of phosphorus, silicon, and potassium, while diminishing Na^+^ absorption, assisting the wheat in mitigating salt stress and enhancing grain production. However, statistically significant superiority over all individual and binary treatments was not consistently established. In another study on wheat subjected to irrigation withdrawal, arbuscular mycorrhizal fungi were combined with *Flavobacterium* and *Pseudomonas* bacteria, along with foliar-applied silicon nanoparticles at concentrations of 30 or 60 mg/L. Variations were observed in phosphorus uptake, water status, fluorescence, and crop yield, which differed depending on the irrigation regime and the specific treatment applied [209]. In contrast, Eliaspour et al. evaluated iron oxide nanoparticles alongside AMF and *Pseudomonas* in drought-stressed maize, although the nanoparticles did not significantly improve the measured traits [210]. The beneficial effects observed in these studies suggest that nanoparticles and beneficial microorganisms may provide complementary forms of protection and benefits to plants, opening a new avenue for investigating the synergistic roles of PGPR, beneficial fungi, and nanoparticles in plant growth, stress tolerance, and agricultural sustainability.

## 7. Challenges and Limitations

Despite research into the potential of PGPR, beneficial fungi, and nanoparticles to support plant growth and minimize the detrimental impacts of abiotic stress, numerous challenges hinder their practical application. Most existing studies have focused on individual components or binary interactions; however, integrated ternary systems comprising PGPR, fungi, and nanoparticles remain insufficiently studied, limiting our understanding of their synergistic mechanisms and field applicability. Furthermore, the efficacy of microbial inoculants and nanoparticles varies significantly depending on plant species, environmental conditions, soil characteristics, and the types and concentrations of nanoparticles employed.

While nanoparticles can enhance nutrient delivery and stress tolerance, excessive concentrations may induce phytotoxic effects, including oxidative stress, impaired root growth, disrupted photosynthesis, and alterations in soil microbial communities. Reports also indicate that high levels of nanoparticles reduce beneficial fungal colonization and impair plant–fungal symbiosis, potentially limiting their positive impact on plant growth and resilience [211,212]. Additionally, the long-term environmental consequences of nanoparticle enrichment in agricultural soil, along with their impacts on food safety, remain poorly understood.

The method of application influences how inoculants are delivered, as well as their survival and stability. PGPR can be applied to seeds, placed in furrows, added via soil irrigation, or used as a root dip. AMF are typically placed in close proximity to developing roots, either through seed treatment or targeted soil application. Nanoparticles can be delivered via seed treatment, foliar spraying, or exposure to the root zone; their uptake depends on the application method and the specific properties of the material used [213,214,215]. No single method is universally optimal for all scenarios [216]. Furthermore, the performance of PGPR and AMF varies based on soil conditions, climate, crop type, and the existing indigenous microbial community. Consequently, candidate organisms that demonstrate local adaptation require functional assessment and field-based efficacy testing at specific sites [217].

Another major limitation is that many studies have been carried out under controlled laboratory experiments or greenhouse conditions, with a few large-scale field studies available to verify their effectiveness in natural environments. Therefore, establishing standardized application protocols and conducting long-term environmental assessments, as well as comprehensive evaluations of their interactions with plants, are essential for the safe and sustainable use of microbial–nanoparticle technologies in modern agriculture.

## 8. Future Perspectives and Conclusions

Although substantial progress has been achieved in understanding the individual and paired applications of PGPR, beneficial fungi, and nanoparticles, studies investigating integrated tripartite systems remain scarce. Therefore, future research should seek to explore and decipher complex interactions among them under diverse environmental conditions. Advanced omics techniques, including genomics, proteomics, transcriptomics, and metabolomics, may provide further insights and contribute to a better understanding of the molecular mechanisms governing these interactions. The development of crop-specific and environmentally safe nano-bioformulations, together with large-scale field validation, will be essential for practical agricultural applications. Moreover, long-term assessments of nanoparticle impact on soil microbial diversity, ecosystem functioning, and food safety are required. Integrating microbial consortia with precision nanotechnology offers a promising pathway toward resource-efficient, climate-resilient, and sustainable agricultural production.

PGPR, beneficial fungi, and nanoparticles independently improve plant growth, nutrient acquisition, and stress tolerance by coordinating diverse physiological, biochemical, and molecular responses. Their paired interactions, including PGPR–fungi, fungi–nanoparticles, and PGPR–nanoparticles, provide additional synergistic benefits by safeguarding osmotic regulation, enhancing photosynthetic efficiency, strengthening antioxidant defense, and stress-responsive signaling pathways. Nevertheless, research addressing their integrated tripartite interactions remains limited. Existing evidence suggests that combining these biological and nanotechnology approaches could substantially improve agricultural productivity under stressful environmental conditions. Future investigations should therefore emphasize the development, optimization, and field validation of tripartite systems to fully exploit their potential for sustainable and climate-smart agricultural systems.

## Figures and Tables

**Table 1:** The combined role of fungi and PGPR in abiotic stress mitigation in plants.

Microbial Inoculants (Species)	Inoculation Dose	Application Method	Experimental Setting	Host Plant	Abiotic Stress	Benefits and Mechanisms	Ref.
*Acaulospora laevis* + *Bacillus subtilis*	NR	NR	Pot experiment	Maize	Drought; 80%, 55%, 35% water levels	↑ Root colonization, microbial biomass carbon, water-use efficiency, root hydraulic conductivity, and photosynthetic rate; enhancing maize grain yield under drought stress.	[149]
AMF consortium + *Bacillus subtilis*/*Bacillus sp*.	Bacteria: 10^9^ CFU/mL; 10 mL per inoculation. AMF: NR.	NR	Field experiment	Maize	Drought; compost-amended field	↑ Root colonization density, the Normalized Difference Vegetation Index (NDVI), total chlorophyll content, and leaf area in maize seedlings. ↑ Water supply and physiological processes, stimulating the accumulation of organic and inorganic osmolytes, and enhancing soil properties (in soil amended with combustion residues), such as cation exchange capacity.	[150]
*Glomus intraradices* + *Bacillus cereus Pb*25	NR	NR	NR	Maize	Heavy metal (Copper) stress; 100 µM	↑ Plant growth and biomass, gas exchange characteristics, nutrient uptake, and gene expression of enzymatic and non-enzymatic compounds. ↓ Oxidative stress and copper accumulation in various plant parts, thereby boosting the maize plant’s tolerance to heavy metal stress.	[151]
*Funneliformis mosseae*/*Claroideoglomus* spp. + *Pseudomonas frederiksbergensis*/*Bacillus tequilensis*/*Burkholderia caledonica* + yeasts	AMF: 100 spores/g substrate; bacteria: 10^8^ CFU/mL.	NR	NR	Strawberry plants	Drought; 85% WHC versus 30% WHC	↑ Biomass, relative water content, number of fruits, photosynthesis rate, transpiration, stomatal conductivity, PSII quantum yield, nitrogen, phosphorus, and potassium concentrations, antioxidant activities, and chlorophyll content, leading to mitigated effects of drought in the inoculated plants.	[152]
AMF + PGPR	NR	NR	Greenhouse trial	Tomato	Drought; WW at 75% FC and WS at 35% FC.	↑ Growth, yield, chlorophyll pigments, fruit quality. ↓ Oxidative stress markers. Dual inoculation mitigated the adverse effects of Phosphogypsum (PG).	[153]
AMF + PGPR	NR	NR	RCBD	Wheat	Drought; WW at 75% FC and d (DS) at 25% FC.	↑ Root dry matter, phosphorus uptake (in combination with compost), auxin content, and plant development. ↑ Antioxidant system, compatible synthesis, and soil glomalin content; leading to ↓ levels of lipid peroxidation and H_2_O_2_, thereby bolstering the plant’s resilience under drought conditions.	[154]
AMF consortium + *Azotobacter chroococcum*, *Bacillus amyloliquefaciens*, *Enterobacter asburiae*	NR	NR	NR	Ashwagandha (Indian ginseng)	Salinity; NaCl; 4.5 dS m^−1^	Salt stress was alleviated by reducing lipid peroxidation and electrolyte leakage, regulating the Na^+^/K^+^ ratio, enhancing withanolide production, and increasing overall growth parameters.	[155]
*Funneliformis mosseae* + *Bacillus amyloliquefaciens*	AMF: 5 g inoculum/hill (approximately 50 spores/g); bacteria: 10^8^ CFU/mL.	Bacterial seed treatment; AMF placed at the planting hill.	NR	Cotton	Drought; irrigation every 30 or 45 days; control: every 15 days	↑Plant height, leaf area, plant dry weight, the number of fruiting branches and bolls, yield and its components, chlorophyll pigments, the efficiency of antioxidant enzymes and the leaf content of total phenolics and proline, thereby boosting the cotton plant’s drought tolerance.	[156]
*Glomus clarum* + *Funneliformis mosseae* + *Gigaspora margarita* + salt-tolerant bacteria	AMF: 5 g soil inoculum + 0.5 g colonized roots. Bacteria: NR.	AMF soil inoculum + colonized roots; bacterial application method: NR.	NR	Maize	Salinity; 0, 50, 100 mM NaCl	↑ Growth parameters, photosynthetic pigments (Chlorophyll a and b, and carotenoids), nutrient content, and antioxidant enzyme activity. ↓ Proline accumulation, Na^+^ uptake, and Na^+^/K^+^ ratio in maize tissues. ↑ Root colonization density and bacterial populations in the rhizosphere. Combined application has a greater impact than individual ones under salinity stress conditions.	[157]
*Trichoderma longibrachiatum* M + *Priestia megaterium* A1/*Bacillus aryabhattai* A2/*Pantoea pleuroti* B3/*Enterobacter kobei* E2	NR	NR	Controlled soil experiment	Rice	Salinity; Saline–alkali soil	↑ Soil microbiome structure, enriching beneficial species, nutrient availability, which aided in mitigating saline–alkali stress in rice.	[158]
*Pseudomonas fluorescens* + *Azospirillum lipoferum* + *Funneliformis mosseae*	NR	NR	NR	Lemon balm	Drought; 50% FC; control: 100% FC	↑ Water status, antioxidant defenses, biosynthesis of rosmarinic acid. ↓ Levels of lipid peroxidation and H_2_O_2_.	[159]
*Rhizophagus irregularis* + *Bacillus subtilis*	AMF: approximately 200–300 spores/plant; bacteria: approximately 10^8^ CFU/mL.	Root-zone drench	Greenhouse experiment	Maize	Drought; greenhouse	↑ Water status, gas exchange, antioxidant regulation, and yield stability and ↓ oxidative damage, leading to mitigation of the effects of drought on maize plants.	[160]
*Funneliformis mosseae* + *Pseudomonas putida*	NR	NR	NR	Citrus	Drought; 45% FC	↑ Antioxidant enzyme activity and modulating gene expression to boost drought resistance. ↑ Stomatal conductance, leaf water potential, carbon uptake, Chlorophyll stability, nutrient uptake, and osmotic adjustment. ↓ Lipid peroxidation and the accumulation of ROS.	[161]
*Glomus etunicatum*/*Funneliformis mosseae* + *Brevibacterium frigoritolerans* KF58B/*Paenibacillus xylanilyticus* KF63C	NR	NR	Controlled conditions	Triticale	Salinity; 0, 100, 200 mM NaCl	↑ Salt stress tolerance during the early seedling stage supported by enzymatic activity observations for stress mitigation.	[162]
Nine-species AMF consortium + four-species bacterial consortium (Medbio)	AMF: approximately 1000 spores/plant; bacterial consortium: 1 mL/L nutrient solution and 1 mL/L foliar spray weekly.	Nutrient-solution application + weekly foliar spray.	Climate chamber	Pepper Plants	Salinity; 200 mM NaCl	↑ Plant performance, relative water content, nutrient uptake, and membrane stability. ↑ Potassium retention and ↓ the accumulation of sodium and chloride, thereby mitigating ionic imbalances. ↑ Chlorophyll content and water relations, photosynthesis, and plant resilience.	[163]

NR = not reported in the available source information or not accessible. Application method is listed only when specified in the table. AMF = arbuscular mycorrhizal fungi; PGPR = plant growth-promoting rhizobacteria; CFU = colony-forming units; FC = field capacity; WW = well-watered; WS = water-stressed; DS = drought-stressed; WHC = water-holding capacity; RCBD = randomized complete block design. The symbols ↑ and ↓ indicate reported increases or decreases relative to the reference group in the study.

**Table 2:** Beneficial activities exerted by fungi and nanoparticles on crops under abiotic stress conditions.

Microbial/Nanoparticle Treatment	Dose	Application Method	Experimental Setting	Host Plant	Abiotic Stress	Benefits and Mechanisms	Ref.
*Funneliformis mosseae* + ZnO nanoparticles	AMF: 5 g/kg soil; ZnO: 1000 ppm (10–30 nm).	AMF soil application; foliar ZnO application.	Pot experiment	Moldavian dragonhead or balm	Salinity; 0, 50, 100 mM NaCl	↑ Dry weight and essential-oil traits (biosynthesis and accumulation); altered ion balance (Na^+^ homeostasis). Co-inoculation stimulated growth and stress tolerance.	[178]
AMF + iron oxide (Fe_3_O_4_) seed coating	AMF dose: NR; Fe_3_O_4_ dose: NR.	Fe_3_O_4_ seed coating; AMF method NR.	NR	Maize	Drought; 100% and 60% field capacity	↑ Photosynthetic rate and leaf relative water content; ↓ Proline.	[179]
Myco-Root AMF blend + ZnO nanoparticles	AMF dose: NR; ZnO: 200 mg/L.	NR.	Two-year experiment; setting: NR	Soybean	Drought; MAD20, MAD50, MAD80 irrigation regimes	↑ Crop productivity, oil quality, physiological traits, mycorrhizal colonization, and seed yield; ↑ nitrogen, phosphorus, potassium, and zinc uptake and antioxidant–enzyme activity; ↓ oxidative-stress markers.	[180]
AMF + rosemary oil encapsulated in nanochitosan	AMF, rosemary oil, and nanochitosan doses: NR.	NR.	Two-year field experiment	Camelina	Drought; contrasting irrigation regimes	↑ Mycorrhizal colonization, Camelina oil production, enzymatic antioxidant defenses, chlorophyll fluorescence, nutrient availability, and antioxidant metabolites (phenolics, flavonoids, tocopherols, ascorbic acid, soluble sugars, and proline); ↓ oxidative stress.	[181]
*Trichoderma koningiopsis*-synthesized iron oxide (Fe_2_O_3_) nanoparticles	Fe_2_O_3_ dose: NR; fungal strain/dose: NR.	NR.	Greenhouse pot experiment	Woody plants	Drought	↑ Growth, physiological traits, and ultrastructure in industrial woody plants. ↓ ROS levels and ↑ antioxidant enzyme activities. ↑ Chlorophyll content, biomass, carbon sequestration, and relative water content. ↑ Stomatal conductance. Preserved stomatal and root integrity.	[182]
*Trichoderma harzianum* + TiO_2_ nanoparticles	Fungal and TiO_2_ doses: NR.	NR.	NR	Peppermint	Cadmium stress	↑ Growth of peppermint plants under cadmium stress, nutrient uptake, and cadmium immobilization.	[183]
*Funneliformis mosseae* + TiO_2_ nanoparticles	AMF: 80 g inoculum/planting hole; TiO_2_: 100 mg/L (60–70 nm).	AMF placed in planting hole; foliar TiO_2_ application.	Field experiment; split-plot design	Sage	Drought; 25, 50%, and 75% MAD	↑ Dry matter, water-use efficiency, and essential-oil quantity/quality under water stress conditions.	[168]
*Funneliformis mosseae* + TiO_2_ nanoparticles	AMF: 10 g soil–root inoculum; TiO_2_: 100 mg/kg soil (~68 nm).	AMF soil–root inoculation; TiO_2_ soil application.	Growth-chamber pot experiment	Common bean	Salinity; 100 and 200 mM NaCl	NPs ↑ AMF colonization, dry biomass, N/P/K uptake, catalase and peroxidase activities, and salinity tolerance; ↓ Na^+^ translocation to shoots; NP effect was limited to its impact on symbiotic mycorrhizal fungi, rather than on the bean plants themselves.	[184]
AMF + ZnO nanoparticles	AMF dose: NR; ZnO: 5 or 10 mg/L.	Seed soaking in ZnO solution for 12 h; AMF method NR.	Saline field soil (EC 5.22–5.54 dS/m)	Wheat	Salinity	↑ Photosynthetic pigments, IAA, phenolic content, antioxidant enzyme activity and lipid peroxidation lead to improved salt stress tolerance.	[185]
*Funneliformis mosseae* + TiO_2_ nanoparticles	AMF dose: NR; TiO_2_: 0, 100, 200, or 500 mg/kg soil.	TiO_2_ soil application; AMF method: NR.	Water-regime experiment: 50%, 70%, and 100% soil moisture	Reed	Water stress + nanoparticle stress (50% soil moisture), normal (70%), flooding (100%); TiO_2_ NP exposure)	TiO_2_ nanoparticles acted as an additional stressor. AMF alleviated the effects of nanoparticle and water stress: ↑ root activity, nutrient uptake, antioxidant–enzyme activity, chlorophyll, and osmolyte accumulation; ↓ MDA and leaf ROS.	[186]

Note. NR = not reported in the supplied table information or not accessible. AMF = arbuscular mycorrhizal fungi; ROS = reactive oxygen species; MDA = malondialdehyde; EC = electrical conductivity; MAD = management allowable depletion. The symbols ↑ and ↓ indicate reported increases or decreases relative to the reference group in the study.

**Table 3:** The combined role of NPs and PGPR in abiotic stress mitigation in plants.

Microbial/Nanoparticle Treatment	Dose	Application Method	Experimental Setting	Host Plant	Abiotic Stress	Benefits and Mechanisms	Ref.
*Alcaligenes pakistanensis* NCCP-650 + ZnFe_2_O_4_ nanoparticles	Bacteria: approximately 10^8^ CFU/mL; ZnFe_2_O_4_: 100 mg/L (25 ± 5 nm).	NR.	Greenhouse pot experiment	Common bean	Drought; water withheld for 10 d (60–65% soil moisture) or 20 d (35–40%); irrigated control at 100% field capacity.	↑ Germination, root and shoot growth, leaf area, stem length, and fresh root weight. The treatment mitigated drought-related epidermal damage, promoted stomatal opening, and increased guard-cell dimensions.	[199]
*Citrobacter* sp. NCCP-668 + ZnFe_2_O_4_ nanoparticles	Bacterial dose: NR; ZnFe_2_O_4_: 100 mg/L.	NR.	Pot experiment	Okra	Cadmium; 30 and 60 µM	↑ Germination, vegetative traits, leaf area, stomatal length, and anatomical traits. Cadmium-contaminated soil was remediated, resulting in sustainable production of okra crops.	[200]
*Bacillus cereus* + citrate-stabilized Ag nanoparticles	Bacteria: 10^8^ CFU/mL; 10 or 20 µL soil-applied. AgNPs: 25 or 40 mg/L; 100 mL/pot.	B. cereus soil application; AgNP application route: NR (100 mL/pot reported).	Greenhouse pots; CRD; n = 4	Sorghum	Cobalt stress; 0, 15, or 25 mg Co/kg soil.	↑ Plant growth, biomass, gas exchange characteristics, enzymatic and non-enzymatic compounds, and their respective gene expression levels. ↓ Proline metabolism, oxidative stress, organic acid secretion patterns, and Co accumulation.	[201]
*Bacillus* spp. + graphene oxide	NR.	NR.	Greenhouse; 4 × 2 factorial design	Sugarcane	Drought; 50% field capacity (FC); control at 100% FC.	↑ CO_2_ assimilation, stomatal conductance, leaf water status, antioxidant defenses, osmotic efficiency, nitrogen/phosphorus/potassium uptake, and biomass; ↓ oxidative damage.	[202]
*Glutamicibacter nicotianae* + silicon; Ag nanoparticles as a stressor	Bacterial and silicon doses: NR; AgNP dose: NR.	NR.	NR	Rice	Ag nanoparticle stress	PGPR–silicon treatment mitigated AgNP-induced growth inhibition and oxidative damage; ↑ photosynthetic pigments, antioxidant enzyme activity, and expression of antioxidant genes and auxin-biosynthesis-related genes.	[203]
*Azospirillum lipoferum* SP2 + *Bacillus coagulans* NCAIM B.01123 + *Bacillus circulans* NCAIM B.02324 + *Bacillus subtilis* MF497446 + ZnO nanoparticles	Each bacterial culture: 10^9^ CFU/mL; peat-based seed inoculum: 950 g/ha. ZnO: 500 mg/L (30–70 nm).	Peat-based seed inoculation; foliar ZnO sprays at 40 and 60 days after sowing.	Field experiment; split-block design; n = 4	Wheat	Salinity; saline groundwater irrigation in sodic–saline soil versus freshwater irrigation	↑ Antioxidant–enzyme activity; potassium and nitrogen uptake; zinc content in grain and straw; and soil urease and dehydrogenase activities. ↓ Electrolyte leakage, proline, MDA, and H_2_O_2_.	[204]
*Pseudomonas* sp. + papaya-extract green-synthesized ZnO nanoparticles	Bacterial dose: NR; ZnO: 10 ppm.	NR.	Pot experiment	Wheat	Individual and combined heat + drought	Synergistic actions enhanced wheat tolerance and growth under single and combined heat and drought stress. ↑ Biomass, photosynthetic pigments, nutrients, soluble sugars, protein, and indole-3-acetic acid content, proline, abscisic acid, and antioxidant enzyme production. ↓ Electrolyte leakage, MDA, and H_2_O_2_.	[205]
*Azotobacter chroococcum* SARS 10 + *Pseudomonas koreensis* MG209738 + silica	Bacterial seed carrier: 950 g/ha; foliar silica: 500 mg/L (10–20 nm).	Seed-carrier application; foliar silica spray.	Field experiment	Barley	Saline-water irrigation	↑ Growth, photosynthetic performance, antioxidant defenses, nutrient uptake, soil microbial activity, and grain productivity; ↓ oxidative damage, soil exchangeable Na^+^, H_2_O_2_, lipid peroxidation, electrolyte leakage, and proline under saline irrigation.	[206]
*Pseudomonas* sp. N5.12 metabolites or metabolite-coated Ag nanoparticles	Metabolites: 5 mL/plant. Coated AgNPs: 60 µg/mL (20.71 ± 0.43 nm), 1 mL/plant.	Metabolite soil drench or foliar spray of metabolite-coated AgNPs.	NR	Tomato	Drought; 6 days without irrigation.	↑ Growth, stem length, and fresh and dry weight, redox balance, and oxidative signaling. ↑ Transpiration (without a change in net photosynthesis). Genomic analysis identified chloroplast and mitochondrial gene-expression changes associated with drought resistance.	[207]

NR = not reported in the available source information or not accessible; AMF = arbuscular mycorrhizal fungi; AgNPs = silver nanoparticles; ZnO = zinc oxide; ZnFe_2_O_4_ = zinc ferrite; CFU = colony-forming units; CRD = completely randomized design; FC = field capacity; MDA = malondialdehyde. The symbols ↑ and ↓ indicate reported increases or decreases relative to the reference group in the study.

## Data Availability

No new data were created or analyzed in this study. Data sharing is not applicable to this article.
